# Horizontal Pod Autoscaling in Kubernetes for Elastic Container Orchestration

**DOI:** 10.3390/s20164621

**Published:** 2020-08-17

**Authors:** Thanh-Tung Nguyen, Yu-Jin Yeom, Taehong Kim, Dae-Heon Park, Sehan Kim

**Affiliations:** 1 School of Information and Communication Engineering, Chungbuk National University, Cheongju, Chungbuk 28644, Korea; tungnt@cbnu.ac.kr (T.-T.N.); yujinyeom@cbnu.ac.kr (Y.-J.Y.); 2 Electronics and Telecommunications Research Institute, Daejeon 34129, Korea; shkim72@etri.re.kr

**Keywords:** cloud computing, container orchestration, custom metrics, Docker, edge computing, Horizontal Pod Autoscaling (HPA), Kubernetes, Prometheus, resource metrics

## Abstract

Kubernetes, an open-source container orchestration platform, enables high availability and scalability through diverse autoscaling mechanisms such as Horizontal Pod Autoscaler (HPA), Vertical Pod Autoscaler and Cluster Autoscaler. Amongst them, HPA helps provide seamless service by dynamically scaling up and down the number of resource units, called pods, without having to restart the whole system. Kubernetes monitors default Resource Metrics including CPU and memory usage of host machines and their pods. On the other hand, Custom Metrics, provided by external software such as Prometheus, are customizable to monitor a wide collection of metrics. In this paper, we investigate HPA through diverse experiments to provide critical knowledge on its operational behaviors. We also discuss the essential difference between Kubernetes Resource Metrics (KRM) and Prometheus Custom Metrics (PCM) and how they affect HPA’s performance. Lastly, we provide deeper insights and lessons on how to optimize the performance of HPA for researchers, developers, and system administrators working with Kubernetes in the future.

## 1. Introduction

In recent years, with the rapid emergence of cloud computing and later edge computing, virtualization techniques have become a sensational topic for both academic research and industrial development as they enable Cloud platforms such as Amazon Web Services (AWS) [1], Google Cloud Platform (GCP) [2], Microsoft Azure [3] to achieve elasticity on a large scale [4]. One of the emerging virtualization techniques is containerization technology, in which a lightweight operating system (OS) equipped with ready-to-deploy application components is packaged into a self-sufficient container ready to run on a host machine that supports multi-tenancy [4,5]. In the host system, different containers run together on the same host OS and in the same Kernel, which helps reduce storage requirements and allows them to achieve near-native performance compared to the host OS [6].

As containers can be deployed on a large scale [7], there is a tremendous need for container orchestration platforms that are highly automatic in terms of deployment, scaling, and management. Amongst various orchestration platforms including Docker Swarm [8], Amazon Elastic Container Service (Amazon ECS) [9], Red Hat OpenShift Container Platform (Red Hat OCP) [10], Kubernetes [11] has become the de facto standard for its popularity. It is an open-source platform, on which it is easy to package and run containerized applications, workloads, and services, and provides a framework for operating scalable distributed systems. Moreover, the containerized applications have portability to be run on any type of OSs and cloud infrastructures [12]. Kubernetes uses Docker as a base environment to run portable and self-sufficient containers, whose instantiations is known as Docker images. It provides a control plane to manage and schedule those containers to run on its cluster of host machines, called nodes, based on their available resources and each container’s specific requirements.

In Kubernetes, one of the most important features is autoscaling because it allows containerized applications and services to run resiliently without the necessity of human intervention. There are three types of autoscalers provided by Kubernetes.

- Horizontal Pod Autoscaler (HPA) supports high availability by adjusting the number of execution and resource units, known as pods [11], based on various requirements. When triggered, HPA creates new pods to share the workloads without affecting the existing ones currently running inside the cluster.
- Vertical Pod Autoscaler (VPA) [11] directly changes the specifications, such as requested resources, of pods and maintains the number of working pods. Therefore, it requires restarting these pods and thus disrupts the continuity of applications and services.
- Cluster Autoscaler (CA) [11] increases the number of nodes when it is no longer possible to schedule pods on the existing ones. Currently, CA only works on commercial cloud platforms such as GCP [2] and AWS [1].

To support autoscaling, Kubernetes monitors pods, applications, host machines, and cluster statistics, called metrics. Autoscalers are triggered when these metrics reach certain thresholds. While Kubernetes provides Resource Metrics by default, their monitoring targets are limited to CPU and memory usage of pods and host machines. Therefore, Customizable (or simply Custom) Metrics can be added with the assist of external software to improve the performance and flexibility of HPA. In this paper, we consider Custom Metrics provided by Prometheus [13], an open-source project run by Cloud Native Computing Foundation (CNCF) [14].

There have been several works that aimed to improve the performance of Kubernetes autoscalers. For example, techniques in References [15,16] are proposed to improve CA and VPA and in References [17,18] focused on improving the performance of HPA and resource monitoring. However, they have not delved into the fundamental knowledge. For instance, questions such as “How does Kubernetes HPA react to different types of metrics?”, “What are the effects of different scraping periods of metrics on Kubernetes?” or “Is monitoring only CPU and memory usage enough for HPA?” need to be addressed. Moreover, documents on Kubernetes and its autoscalers can be found on the official website and several other sources on the Internet [11], but they are written on a functional point of view or simply provide only tutorials on how to install and run Kubernetes. There is a lack of comprehensive and fundamental analysis of Kubernetes’s operational behaviors. Therefore, in this paper, we focus on HPA and seek to improve knowledge on the subject with the following contributions:

- Firstly, we evaluate HPA on diverse aspects such as scaling tendency, metric collection, request processing, cluster size, scraping time, and latency with various experiments on our testbed. Our comprehensive analysis of the results provides knowledge and insights that are not available on the official website and other sources.
- Secondly, besides Kubernetes’s default Resource Metrics, we also evaluate HPA using Prometheus Custom Metrics. By understanding the difference between two types of metrics, readers can have a much firmer grasp on HPA’s operational behaviors.
- Lastly, we provide practical lessons obtained from the experiments and analysis. They could serve as fundamental knowledge so that researchers, developers, and system administrators can make informed decisions to optimize the performance of HPA as well as the quality of services in Kubernetes clusters.

The rest of this paper is organized as follows. Section 2 discusses the existing literature regarding Kubernetes and autoscaling research. Section 3 analyzes Kubernetes’s architecture while Section 4 thoroughly discusses HPA, different metrics, and the use of Readiness Probe, which helps the readers have a better understanding of Kubernetes and HPA before going into details with performance analysis. Section 5 derives lessons on the performance of HPA from the results of diverse experimental scenarios. Lastly, Section 6 concludes the paper.

## 2. Related Work

Kubernetes was originally developed by Google and later transferred to the CNCF [14] as a solution to deploy, manage, and scale containerized applications efficiently in cloud data centers. However, since it is an open-source project, Kubernetes can be configured and modified to be a solid foundation, on which other platforms that meet specific demands can be built and developed. The authors of Reference [19] argue that the current version of Kubernetes scheduler only takes into consideration the virtualizable physical infrastructure including CPU and memory usage, which makes only logical sense. However, from a corporation’s point of view, to improve the efficiency of data centers, other conditions such as geographic location, power infrastructure, and business processes also need to be considered. Thus, the authors propose an enhanced scheduler called Edgetic, which forecasts the optimal placement of pods in terms of performance and power consumption.

In Reference [15], Thurgood and Lennon discuss a number of scenarios including a smart-home environment where there is a great number of input devices while the number of users such as family members frequently fluctuates. This presents the need for HPA and later CA when all the existing nodes are busy. However, currently, default Kubernetes CA is only provided by cloud platform providers such as GCP [2], thus they propose an elastic CA solution for Kubernetes called Free/Open-source Software (FOSS). This solution employs VMware ESXi hosts as nodes and VM tools including vCenter, Foreman for CA operations. Specifically, the vCenter server creates a VM alarm when a CPU or memory threshold is reached at any node, which then executes a bash script creating new VM nodes through Foreman.

In Reference [16], the authors propose a non-disruptive VPA solution called Resource Utilization Based Autoscaling System (RUBAS) incorporating container migration. They argue that resource can be overestimated, which leads to poor utilization rate. Thus, RUBAS calculates the actually required resource for VPA. Moreover, the authors try to tackle the issue of having to restart pods and containers in VPA by creating a checkpoint image using Checkpoint Restore in Userspace (CRIU). Rossi [20] proposes a reinforcement learning model for both horizontal and vertical autoscaling. It aims to ensure the required response times of applications. The authors of Reference [21] develop a hybrid adaptive autoscaler, Libra. It also considers the optimal resource allocation for applications incorporating conventional HPA. Libra is essentially a control loop of VPA and HPA. In the first phase, Libra calculates the appropriate CPU limit with a canary application and based on this new CPU limit, adjusts the number of pods for production applications. After that, if the load reaches the limit, the loop is repeated.

In References [22,23], the authors argue that Kubernetes is currently using relative metrics collected from /cgroup virtual file system through cAdvisor. These metrics can be different from the actual CPU usage in the processors, which can be collected from the /proc file system. This dissimilarity can cause underestimation of required resources. Therefore, the authors propose a correlation model between relative and absolute metrics for CPU-intensive applications, which is employed to correct relative metrics collected by Kubernetes to improve the performance of HPA. With a similar aim in mind, the authors of Reference [24] propose several influencing factors such as the conservative constant, which practically creates a buffer zone for metric fluctuation. Only when the metric value is out of this zone, do HPA actions happen. Another factor is the adaptation interval between successive scaling actions. This reduces unnecessary scaling when the metric value fluctuates.

In References [25,26], the authors apply Kubernetes into resource provisioning for containerized fog computing applications. A network-aware scheduling algorithm that takes into account the network infrastructure such as nodes’ CPU and RAM capacities, device types, and geographic locations to make provisioning decisions is proposed. This algorithm, for example, can consider the round trip delay when scheduling instances of time-critical applications. Another Kubernetes-based fog computing platform that manages geographically distributed containers is proposed in Reference [27]. In this paper, the authors design a service called Autoscaling Broker (AS Broker) to get raw metrics and calculate the optimal number of replicas for HPA based on both CPU and memory usage while reducing applications’ response time. In Reference [12], Chang et al. introduce a Kubernetes-based cloud monitoring platform that provides a dynamic resource provisioning algorithm based on resource utilization as well as application QoS metrics. On this platform, resource metrics are collected and displayed using Heapster v.0.19.1 [28], InfluxDB v.0.9.4.1 [29] and Grafana v.2.1.0 [30], while applications’ response time is calculated with Apache JMeter [31]. This data is aggregated and input into a provisioning algorithm that essentially calculates and fetches the number of pods. Jin-Gang et al. propose a predictive HPA algorithm for a unified communication server using Docker in Reference [18]. The reactive scaling part of this algorithm is the same as Kubernetes’s current algorithm. On the other hand, the algorithm also employs an auto-regressive integrated and moving average (ARIMA) model to predict the future workload, or the number of HTTP Requests [32] to trigger HPA and up-scales beforehand.

Another use-case for Kubernetes HPA is on API gateway systems as proposed in Reference [17]. This system is aimed to simplify internal connection to backend services. Since both the frontend and backend services’ pods are subjects to horizontal autoscaling when necessary, their interconnections can also increase significantly leading to the need for scaling of the gateway system as well. In this work, the authors employ Prometheus custom metrics for HPA operations. However, which metrics and how they are used are not mentioned. In much the same way, Dickel, Podolskiy and Gerndt [33] propose applying Kubernetes HPA on stateful IoT gateways. While stateless applications such as HTTP can be horizontally-scaled easily, stateful ones including WebSocket and MQTT require more attention. For example, after HPA, there will be multiple gateway instances in the cluster. In the publish-subscribe model over information-centric IoT networks, clients (subscribers) and servers (publishers) are required to be connected through the same gateway. Thus, the authors design a framework for IoT gateways using WebSocket and MQTT protocols focusing on establishing and monitoring active connections between clients and servers. The paper also mentions utilizing Prometheus Operator’s custom metrics [34] for HPA. However, similarly to the previously mentioned work, it does not give specific information on how these metrics are collected, calculated, and fetched to HPA-controlling entities.

It is important to note that while the majority of the aforementioned works study Kubernetes and its feature HPA, all of them failed to describe in detail the working principles of HPA and its behaviors when being used with different types of metrics or under various scaling configurations such as the scraping time. This is important for efficient development and management of containerized applications in Kubernetes. Thus, in this paper, we first discuss the architecture of Kubernetes, its components and their inter-communications to establish a solid foundation on the subject, which will be helpful for the readers to have a firm grasp of HPA-related concepts, such as methods of collecting different types of metrics, which will be explained subsequently. As far as we are aware, our paper is the first to achieve such a task. Lastly, we rigorously experiment on a wide range of scenarios to evaluate and analyze diverse aspects of the performance of HPA. Based on the analysis, we provide deep insights and make suggestions on how to optimize Kubernetes HPA to help researchers, developers and sysadmins make informed decisions.

## 3. Architecture of Kubernetes

In this section, we first describe the architecture of a Kubernetes cluster—the main components and their intercommunication inside the cluster. Then, we take a closer look into how Kubernetes’s services enable applications currently running on pods within the cluster to work as a network service.

### 3.1. Kubernetes Cluster

**Figure 1:**
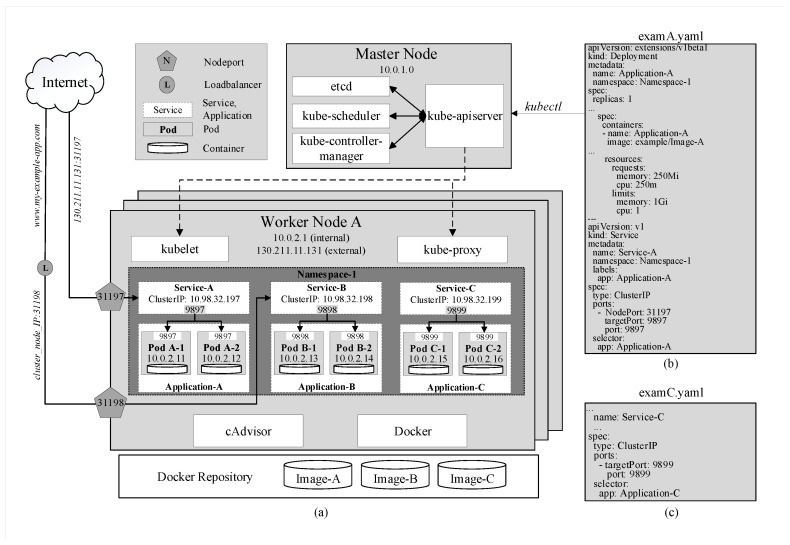
(**a**) The architecture of Kubernetes. (**b**,**c**) Examples of YAML code for application deployment in Kubernetes.

As shown in Figure 1a, each Kubernetes cluster consists of at least one master node and several worker nodes. In practice, it is possible to have a cluster with multiple master nodes [11] to ensure high availability of the cluster by replicating the master, so in cases where one of the masters fails, a quorum still exists to run the cluster.

The most basic execution and resource unit in Kubernetes is called a pod, which contains a container or a group of containers and instructions on how these containers should be operated. Each pod represents an instance of an application and always belongs to a namespace. Furthermore, pods that belong to the same application are identical and have the same specifications. In this sense, a pod can be referred to as a replica as well. Upon the deployment of an application, the desired number of replicas, as well as the amount of requested resource, need to be specified. Figure 1b shows the application is created under the name Application-A in Namespace-1 and requests for each of its pods 250Mi and 250 m, of memory and CPU available. “Mi” denotes “Mebibyte” and “m” denotes “millicore”—a unique unit equal to 1/1000 of a CPU core. It is defined by Kubernetes as a granular way to measure the CPU resource so that more than one pod can share a CPU core.

Moreover, each pod is assigned with a unique IP address [11] within the cluster as shown in Figure 1a. This design allows Kubernetes to scale applications horizontally. For example, when an application requires more computational resources, instead of having to adjust the specifications of the existing pods, users can simply create another identical pod to share the load. This additional pod’s IP address will then be included in the application’s service that routes incoming traffic to the new pod as well as the existing ones. This will be discussed again in more detail.

#### 3.1.1. Master Node

The master node has total control over the cluster through four main components of the Control Plane, namely kube-apiserver, kube-controller-manager, kube-scheduler, and etcd [11] as shown in Figure 1a.

- kube-controller-manager watches over and ensures that the cluster is running in the desired state. For instance, an application is running with 4 pods; however, one of which is evicted or missing, kube-controller-manager has to ensure that a new replica is created.
- kube-scheduler looks for newly created and unscheduled pods to assign them to nodes. It has to consider several factors including nodes’ resource availability and affinity specifications. In the previous example, when the new pod has been created and currently unscheduled, kube-scheduler searches for a node inside the cluster that satisfies the requirements and assigns the pod to run on that node.
- etcd is the back storage that has all the configuration data of the cluster.
- kube-apiserver is the foundational management component that can communicate with all other components and every change to the cluster’s state has to go through it. kube-apiserver is also able to interact with worker nodes through kubelet, which will be discussed subsequently. Moreover, users can manage the cluster through the master by passing kubectl commands to kube-apiserver. In Figure 1, after running the command *kubectl apply -f examA.yaml*, the specifications in this file are passed through kube-apiserver to kube-controller-manager for replica-controlling and to kube-scheduler for scheduling pods on specific nodes. They will reply to kube-apiserver who will then signal to these nodes to create and run the pods. These configurations are stored in etcd as well.

#### 3.1.2. Worker Node

Worker nodes allocate computing resources in the form of pods and run them according to the instructions from the master.

- kubelet is a local agent that operates the pods as instructed by the master node’s kube-apiserver and keeps them healthy and alive.
- kube-proxy (KP) allows external and internal communication to pods of the cluster. As mentioned earlier, each pod is assigned a unique IP address upon creation. This IP addresses are used by KP to forward traffic from within and outside of the cluster to pods.
- Container Run-time: Kubernetes can be thought of as a specialized orchestration platform for containerized applications and thus requires container runtimes in all nodes including the master to actually run the containers. It can run on various runtimes including Docker, CRI-O and Containerd. Amongst those, Docker [8] is considered the most common one for Kubernetes. By packaging containers into lightweight images, Docker allows users to automate the deployment of containerized applications.
- CAdvisor (or Container Advisor) [35] is a tool that provides statistical running data of the local host or the containers such as resource usage. This data can be exported to kubelet or managing tools such as Prometheus for monitoring purposes. CAdvisor has native support for Docker and is installed in all nodes along with Docker to be able to monitor all nodes inside the cluster.

### 3.2. Kubernetes Service

In Kubernetes, it is possible to access each pod internally because it has a unique IP address that can be accessed inside the cluster. However, since pods can be created and die at any moment, using individual pods’ IP addresses is not a plausible solution. Additionally, these IP addresses cannot be accessed from outside the cluster, which renders user requests or communications between applications deployed in different clusters impossible.

A solution to these cases is Kubernetes Service [11], which is an abstract object that exposes a set of pods to be easily accessed both internally and externally. There are three types of Kubernetes Service:

- ClusterIP is assigned to a service upon creation and stays constant throughout the lifetime of this service. ClusterIP can only be accessed internally. In Figure 1a, services A, B, and C are assigned with three different internal IP addresses and expose three service ports 9897, 9898, and 9899, respectively. For example, when the address 10.98.32.199:9899, which consists of the cluster IP and exposed port of Service-C, is hit within the cluster, traffic is automatically redirected to targetPort 9899 on containers of pods of Application-C as specified in the YAML file by the keyword selector in Figure 1c. The exact destination pod is chosen according to the selected strategy.
- NodePort is a reserved port by the service on each node that is running pods belonging to that service. In the example in Figure 1b, NodePort 31197 and service port 9897 are virtually coupled together. When traffic arrives at NodePort 31197 on node A, it is routed to Service A on port 9897. Then, similarly to the previous example, the traffic is, in turn, routed to pods A-1 and A-2 on targetPort 9897. This enables pods to be accessed from even outside the cluster. For instance, if node A’s external IP address 130.211.11.131 is accessible from the internet, by hitting the address 130.211.11.131:31197, users are actually sending requests to pods A-1 and A-2. However, it is obvious that directly accessing nodes’ IP addresses is not an efficient strategy.
- LoadBalanceris provided by specific cloud service providers. When the cluster is deployed on a cloud platform such as GCP [2], Azure [3] or AWS [1], it is provided with a load balancer that can be easily accessed externally with a URL (www.my-example-app.com). All traffic to this URL will be forwarded to nodes of the cluster on NodePort 31198 in a similar manner to the previous example as illustrated in Figure 1a.

## 4. Horizontal Pod Autoscaling

**Figure 2:**
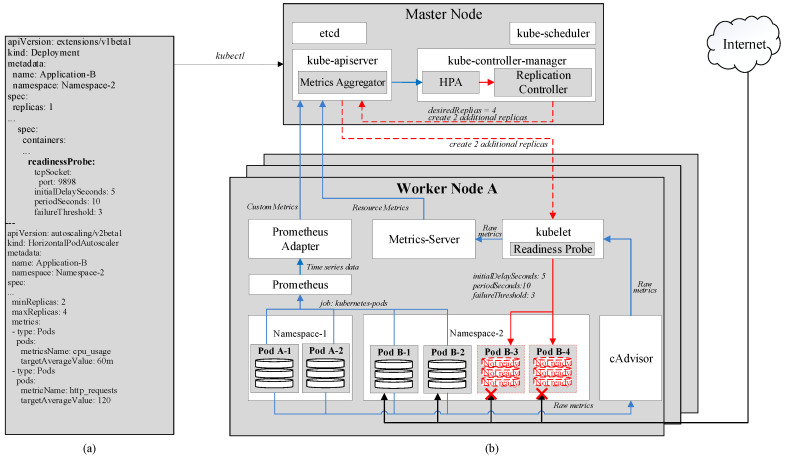
(**a**) Examples of YAML code for configuring HPA and Readiness Probe in Kubernetes. (**b**) Horizontal Pod Autoscaling’s (HPA) architecture.

In Kubernetes, HPA is a powerful feature that automatically raises the number of pods, to increase the application’s overall computational and processing power, without having to stop the application’s currently running instances [11]. Once successfully created, these new pods are able to share the incoming load with the existing ones. From a technical point of view, HPA is a control loop implemented by kube-controller-manager. By default, every period of 15 s, also known as sync cycle, kube-controller-manager compares the collected metrics against their thresholds specified in HPA configurations. Figure 2a shows the configurations of an HPA. “minReplicas” and “maxReplicas” refer to the minimum and maximum numbers of pods that should be running in the cluster. In the example, minReplicas and maxReplicas are 2 and 4, respectively. The Replication Controller, which is a component of kube-controller-manager, keeps track of the replica set and ensures that no less than 2 and no more than 4 pods of this application run in the cluster at all times. The metric used for this HPA is CPU usage. Once the average value of CPU utilization reaches a preset threshold, HPA automatically increases the number of pods as by calculating the following

(1) $$desiredReplicas=\lceil currentReplicas*\frac{currentMetricValue}{desiredMetricValue}\rceil ,$$

where *desiredReplicas* is the number of pods after scaling, *currentReplicas* is the number of pods currently running, *currentMetricValue* is the latest collected metric value, *desiredMetricValue* is the target threshold. *desiredMetricValue* is actually the threshold *targetAverageValue* in Figure 2a.

In the example, when the *currentMetricValue*, which in this case is CPU usage, hits 150 m, which is higher than the threshold *desireMetricValue* of 60 m, the *desiredReplicas* equals to 2×(150/60), or 5. However, as the maximum number of replicas is only 4, kube-controller-manager only signals to kube-apiserver to increase 2 more replicas. After this, if the average CPU usage declines to 40 m, the *desiredReplicas* is 4×(40/60), or 3. Therefore, one of the newly created pods will be removed. However, it is worth noting that to avoid thrashing from creating and removing pods repeatedly as the metrics can fluctuate significantly, each newly created pod is kept running for at least a downscale delay period before it can be removed from the cluster. This period is set at 5 min [11]. Furthermore, it is possible for HPA to use several metrics, each of which has its own threshold. When any of these metrics reaches its threshold, HPA scales up the cluster in the above-mentioned manner. However, for scaling-down operations, all of these metrics are required to be below their thresholds.

The above-mentioned metrics used by kube-controller-manager for horizontal autoscaling are either Kubernetes’s default Resource Metrics or external Custom Metrics provided by Prometheus [13], Microsoft Azure [3], et cetera. In the scope of this paper, we only discuss Kubernetes Resource Metrics and Prometheus Custom Metrics, which are the most popular for HPA.

### 4.1. Kubernetes Resource Metrics

As shown in Figure 2b, cAdvisor acts as a monitoring agent to collect core metrics, such as CPU, memory usage, of the host machines and running pods, and publish these metrics through an HTTP port. For example, in Figure 2b, cAdvisor is currently monitoring 4 existing pods A-1, A-2, B-1, and B-2. With the help of kubelet, the Metrics-Server scrapes these metrics periodically. The default scraping period is 60 s and can be adjusted by changing Metrics-Server’s deployment configurations. Then, the Metrics-Server exposes them to the Metrics Aggregator in kube-apiserver as CPU and memory usage of individual pods and nodes, whose average values will be calculated and fetched to HPA. This is referred to as the resource metrics pipeline [11]. Kubernetes Resource Metrics can be checked manually by passing commands kubectl top pod and kubectl top node to kube-apiserver.

### 4.2. Prometheus Custom Metrics

Prometheus [13] allows flexible monitoring as it exposes monitored targets as endpoints and periodically pulls their metrics through an HTTP server. It can monitor a wide range of targets including nodes, pods, services, or even itself. The monitoring operation for each of these targets is called a job. While Prometheus has its default global scraping period of 60 s, its jobs can have their own scraping periods. For jobs that are too short-lived to be scraped, Prometheus has component called Pushgateway into which these jobs can directly push their metrics once they exit. Then, by exposing the Pushgateway as an endpoint as well, Prometheus can scrape these metrics later even after the jobs have been terminated. As shown in Figure 2b, Prometheus scrapes metrics from existing pods A-1, A-2, B-1, and B-2 with a job called kubernetes-pods.

The scraped data is then stored in the form of time series in the Time Series Database (TSDB), which is exposed to the Prometheus Adapter [36], which is written in PromQL (Prometheus Query Language)—a functional query language [13] and has several queries to actually process raw time series metrics. For example, query *rate(http_requests_total[1m])* returns the per-second average rate of the time series collected within 1 min. The number of the time series depends on the scraping period. A 15-s scraping period would result in a total of 4 time series for the above query.

Once the queries are finished, the resulted metrics will be sent to the Metrics Aggregator in the kube-apiserver and by which fetched to the HPA. While the Metrics-Server can monitor only CPU and memory usage, Prometheus can offer a variety of custom metrics. The metric in the previous example can be employed as the average arrival rate of HTTP requests, which can be added to the HPA in Figure 2a. Therefore, in this case, where there are two input metrics, HPA scales up whenever either one of the metrics has reached its threshold and scales down when both metrics are below their thresholds.

### 4.3. Readiness Probe

In most cases, pods are not ready to serve traffic immediately after their creation as they may have to load data or configurations. Therefore, if traffic is sent to these newly created pods during their startup time, the requests will obviously be failed. As a solution, Kubernetes provides a feature named Readiness Probe [11], which checks the statuses of new pods and only allow traffic to them once they are Ready. In Figure 2a, the initialDelaySeconds, defines the amount of time between the pods’ creation and when they are first checked for readiness. If, after the check, the pods are still not ready, Kubernetes will check again periodically every periodSeconds. In this example, once pods B-3 and B-4 are created, Kubernetes gives it 5 s to startup and get ready. After the first readiness check, if the pod’s status is ready, it will start serving incoming traffic immediately. On the other hand, if it is not, Kubernetes checks every 10 s for 3 more times, which is defined by failureThreshold, before giving up and deciding to reset or deem the pod Unready based on preset configurations.

## 5. Performance Evaluations

In this section, we describe our experimental setup before showcasing and discussing evaluation results in detail to confirm our understanding of Kubernetes and its HPA feature. We also provide analysis and deep insights on how to optimize HPA.

### 5.1. Experimental Setups

We set up a Kubernetes cluster of 5 nodes, consisting of 1 master node and 4 worker nodes, inside a physical machine that runs on Intel(R) Core (TM) i7-8700 @ 3.20Ghz * 12. Each node of the cluster runs a virtual machine with Ubuntu 18.04.3 LTS operating system, Docker version 18.09.7, Kubernetes version 1.15.1. Regarding computing capabilities, the master is allocated 4 core processors and 8GB of RAM, compared to 2 core processors and 2GB of RAM for each of the worker nodes. Moreover, Gatling open-source version 3.3.1 [37] is employed as the load generator that sends HTTP requests to our application through a designated NodePort on each worker node.

Our application is designed to be CPU-extensive. In other words, once it successfully receives a HTTP request, it uses CPU resource until sending back a response to the source. The CPU request and limit of each replica are 100 m and 200 m, respectively. The number of replicas ranges from the minimum of 4 (average of 1 replica/node) to the maximum 24 (average of 6 replicas/node).

All experiments are run for 300 s. During the first 100 s, the average incoming request rate sent by Gatling is approximately 1800 requests/s, while it is roughly 600 requests/s for the next 100 s, which generates a total of 240,000 requests. We define these two periods as high traffic period (HTP) and low traffic period (LTP), respectively. The rest of the simulation time is used for observing the decrease of metrics while there are no arriving requests. In this paper, we test Kubernetes HPA’s performances in 7 different experiments. Each experiment is repeated 10 times to ensure its accuracy.

### 5.2. Experimental Results

#### 5.2.1. HPA Performances with Default Kubernetes Resource Metrics

Setup. Metrics’ scraping period is set at the default value of 60 s.

Goal. We aim to evaluate the performance of HPA using default Kubernetes Resource Metrics (KRM) in terms of CPU usage, numbers of replicas and failed requests under the default scraping period.

**Figure 3:**
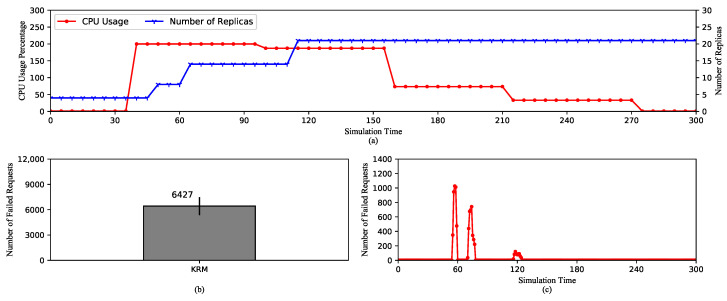
HPA using default Kubernetes Resource Metrics (KRM). (**a**) The average CPU usage and the scaling of the replica set. (**b**) The total number of the failed requests. (**c**) The timeline of the failed requests.

Looking into Figure 3a, the average CPU usage increases to the limit because of the high rate of requests. After that, it decreases as the number of replicas is raised. It can be observed that the most striking point is that the metric value of CPU usage changes every scraping period (60 s). This is because Kubelet only scrapes the raw metrics from cAdvisor at the beginning of a scraping cycle. Then, the metrics are reported without any modification to the Metrics-Aggregator through the Metrics-Server. In other words, the reported values of the metrics are exactly equal to the scraped values.

The first scaling action expanding the replica set to 8 happens around 50th second as a result of the increase in the metric value between 35th and 40th second. After that, as the metric value reaches the maximum of 200%, the replica set is expanded again to 15 at 65th second. However, the third scaling action happens at 110th s, which is 55 s later, even though the CPU usage is still high. This is due to a very important characteristic of HPA. By default, HPA checks the metric value every 15 s. At the time of checking, if there is no change in the value compared to the last check, it is deemed unnecessary to adjust the number of replicas. We can see that from 40th to 100th second, which is exactly one scraping cycle, the CPU usage does not change. After this, it decreases to about 180%, which actually triggers the third scaling action to 21 replicas. Here, the metric value again remains stable until 160th s, where it begins to drop and does not cause any more scaling-ups. The number of replica remains stable until the end of the experiment because HPA has to wait for 5 min from the last scaling-up to scale down. This design aims to avoid thrashing caused by continuous scaling actions.

Figure 3b,c show the number of failed requests and the time of failures. These requests are refused during the scaling operations, because newly created pods are not ready to serve traffic. Any requests routed to them during this time will be failed. Furthermore, we can see that the first scaling-ups cause the majority of failed requests because it happens during the HTP. The high request rate causes more requests to be refused. As opposed to this, the third scaling-up causes only a small number of failures.

In conclusion, it is important to note that Kubernetes HPA is designed to check the metric value periodically and decision-making on scaling depends on whether the metric value is changed compared to the last check. In addition, since KRM’s value is only changed every scraping period, the number of created replicas depends on this factor. Therefore, the next experiment analyzes the effect of the length of the scraping period on HPA’s performance.

#### 5.2.2. HPA Performances with Default Kubernetes Resource Metrics and Different Scraping Periods

Setup. KRM’s scraping period is adjusted to 15 s, 30 s, and 60 s, respectively.

Goal. We aim to investigate the effect of different scarping periods on HPA’s performance.

**Figure 4:**
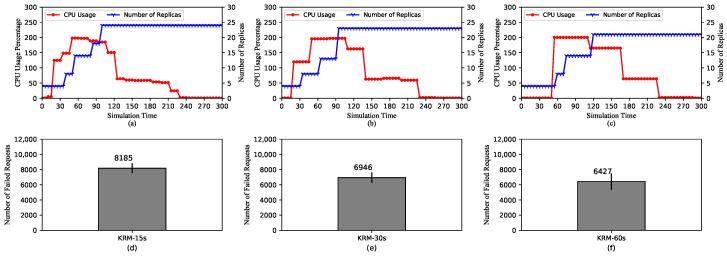
HPA using default Kubernetes Resource Metrics (KRM). (**a**–**c**) The average CPU usage and the scaling of the replica set for scraping periods of 15 s, 30 s, and 60 s, respectively. (**d**–**f**) The total number of the failed requests for scraping periods of 15 s, 30 s, and 60 s, respectively.

Figure 4 shows the performance of HPA using KRM with three different scraping periods of 15 s, 30 s, and 60 s. In Figure 4a–c, we can see that the trend of metric values still follows the pattern described earlier. The values change every scraping period for all three cases. In other words, as the period is lengthened, the metric values remain at the same level for a longer amount of time.

However, the maximum number of replicas tends to decrease as the scraping period increases. In Figure 4a,b, the maximum numbers of replicas are 24 and 23, respectively. On the other hand, for the case of the 60-second scraping period, it is only 21. It is because if the metric value does not change, it would not trigger scaling actions, as stated previously. In this case, because the value changes more frequently for the short scraping periods, the number of replicas is also raised more frequently. Moreover, Figure 4d–f show the numbers of failed requests in three cases. Similar to the maximum number of replicas, that of failed requests tends to decrease with longer scraping periods. This is because the more unready pods are getting incoming traffic, the more requests will be refused. Note that the failure of requests coming to unready pods can be solved with the Readiness Probe, whose effect will be analyzed in Section 5.2.7.

With the above explanations, it can be concluded that when under the same load for the same amount of time, a longer scraping period causes HPA to raise a smaller number of replicas. This comes with pros and cons. On the one hand, a longer scraping period may result in efficient resource allocation by triggering scaling actions relatively slowly adding a smaller number of replicas. On the other hand, it can lead to a lack of required resources if the incoming load becomes too high.

#### 5.2.3. HPA Performances with Prometheus Custom Metrics

Setup. Prometheus Custom Metrics’ scraping period is set at the default value of 60 s.

Goal. We aim to evaluate the performance of HPA using Prometheus Custom Metrics in terms of CPU usage, numbers of replicas and failed requests to compare with HPA using KRM. 

**Figure 5:**
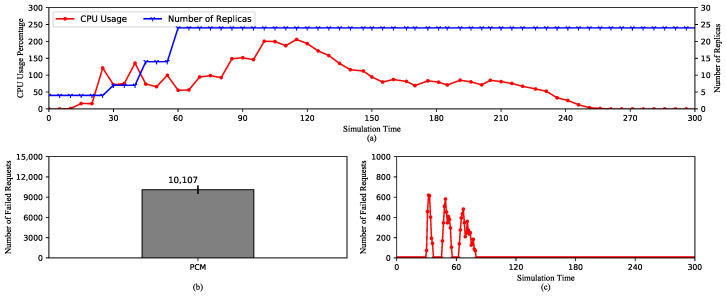
HPA using Prometheus Custom Metrics (PCM). (**a**) The average CPU usage and the scaling of the replica set. (**b**) The total number of the failed requests. (**c**) The timeline of the failed requests.

Looking at Figure 5a, it can be seen that PCM’s metric value changes very frequently, every time it is queried. This is completely contrary to the case of KRM. The reason for this lies in the way PCM is collected. Even though Prometheus also scrapes pods’ metrics according to the scraping period, these metrics have to go through the *rate()* function at Prometheus Adapter, which calculates their per-second average of increase [13]. Moreover, it is important to note that the *rate()* function also performs extrapolation based on the current trend of metrics when necessary such as in cases where time-series data points are missing. Therefore, the CPU usage, in this case, changes every query period. These frequent changes, in turn, cause HPA to increase the number of replicas quickly to the maximum of 24 as opposed to only 21 in the case of KRM. Therefore, PCM offers the advantage of responsiveness to frequent changes in the metric value. Quickly increasing the number of replicas, or overall computational power, allows HPA to deal with surges of incoming loads. However, a disadvantage is a higher number of failed requests as shown in Figure 5b,c.

#### 5.2.4. HPA Performances with Prometheus Custom Metrics and Different Scraping Periods

Setup. Metric scraping period is set 15 s, 30 s and 60 s.

Goal. We aim to investigate the effect of different scarping periods on the performance of HPA using PCM.

**Figure 6:**
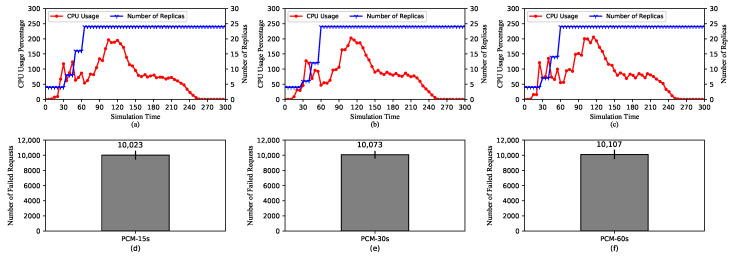
HPA using Prometheus Custom Metrics (PCM). (**a**–**c**) The average CPU usage and the scaling of the replica set for scraping periods of 15 s, 30 s, and 60 s, respectively. (**d**–**f**) The total number of the failed requests for scraping periods of 15 s, 30 s, and 60 s, respectively.

Figure 6 shows the performance of HPA in terms of CPU usage and the number of replicas with three different scraping periods of 15 s, 30 s, and 60 s, respectively. It can be seen that in all three cases, the trends of the graphs are very similar. As explained earlier, the reason is that Prometheus Adapter, the entity responsible for transforming raw metrics into Custom Metrics, can perform extrapolation to provide the metrics when raw data points are missing. If the moment of querying is in the middle of a scraping cycle, Prometheus Adapter, the rate() in particular, calculates the metrics based on the previously collected data points. As a result of the similarity in metric values, the numbers of replicas of three cases increase in a similar pattern during the same period.

We can conclude that PCM is not as strongly affected by adjustments to the scraping period as KRM. A longer scraping period can be chosen as it will reduce the amount of computational and internal communicational resources required to collect and pull the metrics. However, it is worth noting that longer scraping periods mean there are fewer data points, which can reduce the precision of the *rate* function.

#### 5.2.5. Comparison of HPA Performances in a 2-Worker Cluster and a 4-Worker Cluster

Setup. Two clusters of 2 and 4 worker nodes are set up. Worker nodes are identical and the maximum and minimum numbers of replicas are the same for both cases.

Goal. We aim to investigate and compare the performances of HPA in these two cases.

**Figure 7:**
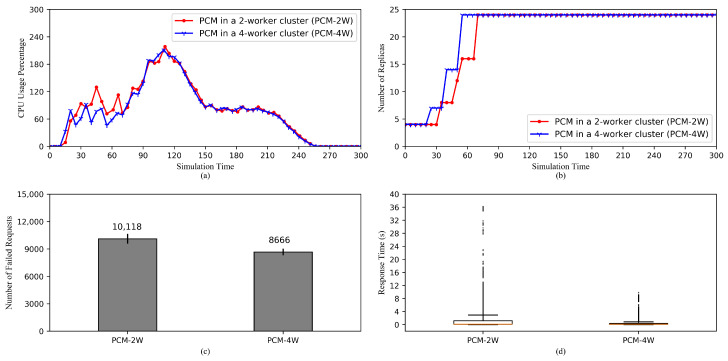
Comparisons of HPA performances in 2-worker and 4-worker clusters. (**a**) The average CPU usage. (**b**) The scaling of the replica set. (**c**) The total number of the failed requests. (**d**) Response time of the successful requests.

Figure 7 shows the performance comparison between HPAs using PCM in a 2-worker cluster and a 4-worker cluster (PCM-2W and PCM-4W). From Figure 7a, it can be concluded that the general trends of two CPU usage values are largely similar. The only noticeable differences in the CPU usage appear between 30th and 70th s. This is a result of the differences in the increases of replicas as shown in Figure 7b, where PCM-2W increases the number of replicas slower than PCM-4W does. This is because while the maximum number of replicas is 24 for both HPAs, the average numbers of pods on each node are 6 and 12 for the 4-worker and 2-worker clusters, respectively. It indicates that a node in the 2-worker cluster has to process a higher computation load when scaling, which makes creating and allocating resources for additional pods slower compared to a node in the 4-worker cluster.

Moreover, Figure 7c shows that the number of failed requests of PCM-2W is higher than that of PCM-4W. This is because newly created pods take longer to get ready in the case of PCM-2W. However, since the trends of increases in two cases are generally resembling each other, the difference is relatively moderate.

Furthermore, it is understandable that when the requests reach the 2-worker cluster’s nodes, they would have to stand in a much longer queue than they would in the case of the 4-worker cluster. This is the main reason why the response time of PCM-2W is remarkably longer than that of PCM-4W in Figure 7d. In addition, the difference in the speeds of pod creation in two cases also contributes to this contrast.

#### 5.2.6. Comparison of HPA Performances with Different Custom Metrics

Setup. As opposed to KRM, which can monitor only CPU and memory usage, PCM can monitor other metrics such as the rate of incoming HTTP requests. In the first case, only the request rate is used for HPA. On the other hand, in the second case, it is combined with CPU usage.

Goal. We aim to investigate the effect of the use of different custom metrics on HPA’s performance.

**Figure 8:**
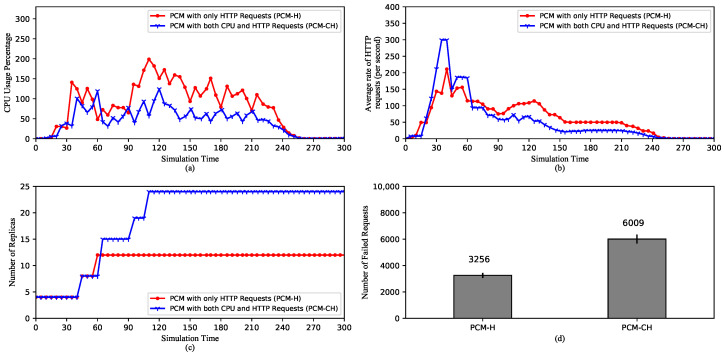
Comparison of HPA Performances with different custom metrics. (**a**) The average CPU usage. (**b**) The average rate of HTTP requests. (**c**) The scaling of the replica set. (**d**) The total number of the failed requests.

Figure 8 shows the comparison between performances of HPA using HTTP requests (PCM-H) and HPA using CPU and HTTP requests (PCM-CH). PCM-H provides autoscaling based on the incoming request rate. On the other hand, PCM-CH combines both the rate and the CPU usage. When multiple metrics are specified, HPA scales up if either one of the metrics reaches its threshold. From the CPU usage comparison in Figure 8a, we can see that in general, CPU usage of PCM-H is significantly higher than that of PCM-CH in general. While in Figure 8b, PCM-CH’s average request rate is considerably higher than PCM-H’s average request rate from 30th to 60th second. After that, it remains lower until the 250th second. This has resulted from the fact that PCM-CH’s request rate rises sharply, which triggers the first scaling reactions, as shown in Figure 8c, after which the CPU usage is still higher than its threshold and causes subsequent rises to the maximum number of replicas. Here, because PCM-CH has more replicas, so the average request rate is lower. On the other hand, PCM-H uses only the request rate. After the first scale, the rate decreases and remains below the threshold, which does not trigger any more scaling-up. Moreover, because now it has only 13 replicas, the CPU usage rises and stays at a high level. Furthermore, since there are fewer increases of pods, PCM-H produces only approximately half the number of failed requests of PCM-CH.

#### 5.2.7. Comparison of HPA Performances with and without Readiness Probe

Setup. Regular HPA is set up in one case while it is accompanied by Readiness Probe in the other.

Goal. We aim to investigate the use of Readiness Probe, its effect on the number of failed requests and response time.

**Figure 9:**
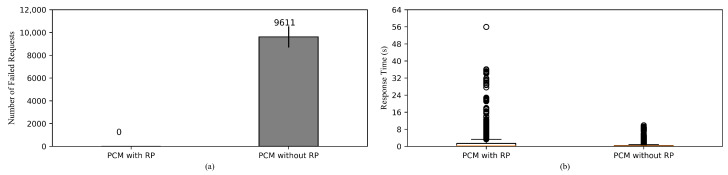
Comparison of HPA performances with and without Readiness Probe. (**a**) The total number of the failed requests. (**b**) Response time of the successful requests.

Figure 9a shows the comparison between HPA coupled with the use readiness probe (RP) and regular HPA in terms of numbers of failed requests. It is obvious that in the case of using RP, there are no failed requests, because when the additional pods are getting ready, Kubernetes service does not route any traffic to them. Only once they have been deemed ready, they can receive and process incoming requests. Therefore, we can expect that the failed requests observed from the previous experiments can be avoided by using RP. However, this is rather a tradeoff, as the general response time is significantly higher than the case of Not using RP as shown in Figure 9b, since more traffic is routed to the existing pods.

### 5.3. Discussion

To summarize the previous experiments and analyses, we list out a few key points on Kubernetes HPA’s behaviors.

- On KRM and PCM: KRM only reports values of metrics and is able to change only once every scraping cycle as opposed to PCM, which are able to maintain the trend of metrics’ values even during the middle of a scraping cycle or if there are missing data points. As a direct consequence, KRM expands the replica set slower and mostly to a smaller number of replicas compared to PCM. The advantage of this behavior is obviously less resource consumption. On the other hand, when under high load pods could be crashing or becoming unavailable. Therefore, we suggest KRM for applications with more stable loads such as video processing services. In this case, the number of requests from viewers is usually small as it takes at least a few minutes to a few hours for a video. On the contrary, PCM is more suitable for applications with frequent changes in metrics. For instance, e-commerce websites may experience continuous surges in a few hours during a sale event, thus require fast system reactions.
- On the scraping period: Adjustments to the scraping period of PCM do not strongly affect the performance of HPA. Therefore, a longer period can be set to reduce the amount of resource used for pulling the metrics. However, it is worth noting that a overly long period can cause imprecision in calculating the metrics. Regarding KRM, the scraping period has a significant influence on the performance of HPA. A longer period can reduce the amount of resource allocated for new pods, but it can cause decreased quality of service. Therefore, the scraping period should be carefully chosen having considered the type of service and the capability of the cluster.
- On the cluster size: It is obvious that a 4-worker cluster has more computational power, which allows it to perform HPA operations faster, than a 2-worker cluster, assuming workers of the two clusters are identical in terms of computational capabilities. In addition, the communicational capability of the 4-worker cluster is superior to the 2-worker cluster. This results in the difference in request response time of the two clusters. However, even if the 2-worker cluster has equal computational and communicational power, it is safer to spread pods to a wider cluster as half of the pods can become unavailable when a node crashes, compared to a forth of the pods in the case of the 4-worker cluster.
- On HPA with different custom metrics: Prometheus enables the use of custom metrics such as HTTP request rate to meet specific demands. Especially combining multiple metrics together can also increase the effectiveness of HPA as changes in any individual metric will cause scaling reactions. However, as a downside, this may result in waste of resource. Therefore, metrics, or combinations of metrics, should be chosen according to the type of the application. For instance, a gaming application may have various request sizes. Requests to move a character around the map are small in size but their number can be numerous. Thus, the request rate should be considered, so that each request can be quickly served, which reduces the “lagging” effect and improves the overall gaming experience. On the other hand, requests to load new locations’ maps are heavy but small in number. Here, computational requirements grow significantly higher, which indicates HPA should scale based on CPU and memory usage. In short, Custom Metrics enable applications to consider various factors such as the number of requests, latency, and bandwidth for efficient horizontal autoscaling.
- On Readiness Probe: It is a powerful feature from Kubernetes to prevent requests from being routed to unready pods, which will reject the requests. However, routing a number of requests to existing pods can cause the rest of the requests to have significantly longer response time. Therefore, between keeping the incoming requests alive or letting them fail and expecting re-requests, one should be chosen carefully based on balancing between system resources and QoS requirements.

## 6. Conclusions

Kubernetes is a powerful orchestration platform for containerized applications and services, and can be applied into important future technologies including cloud/edge computing and IoT gateways. Its feature HPA provides dynamic and effective scaling for applications without the necessity of human intervention. In this paper, we have given the first comprehensive architecture-level view of both Kubernetes and HPA. How each type of metrics, including Kubernetes Resource Metrics and Prometheus Custom Metrics, are collected, calculated and fetched to HPA was also thoroughly explained. Moreover, we conducted several experiments covering a variety of scenarios and provided clear analysis for the behaviors of Kubernetes HPA.

This paper should serve as a fundamental study for further research and development of Kubernetes and HPA. In the future, we aim to expand our experiments with more HPA scenarios as well as to develop a more efficient scaling algorithm for Kubernetes.
